# Neutrophil Extracellular Traps (NETs) in Cancer Invasion, Evasion and Metastasis

**DOI:** 10.3390/cancers13174495

**Published:** 2021-09-06

**Authors:** Urszula Demkow

**Affiliations:** Department of Laboratory Diagnostics and Clinical Immunology of Developmental Age, Medical University of Warsaw, 02-091 Warsaw, Poland; urszula.demkow@wum.edu.pl

**Keywords:** cancer, neutrophil extracellular traps, metastasis, evasion

## Abstract

**Simple Summary:**

This review focuses on the pro-tumorigenic action of neutrophil extracellular traps (NETs). NETs were found in various samples of human and animal tumors. The role of the NETs in tumor development increasingly includes cancer immunoediting and interactions between immune system and cancer cells. NETs awake dormant cancer cells, play a key regulatory role in the tumor microenvironment, and exacerbate tumor aggressiveness by enhancing cancer migration and invasion capacity. Furthermore, NETs induce the epithelial to mesenchymal transition in tumor cells. NET proteinases can also degrade the extracellular matrix, promoting cancer cell extravasation. Moreover, NETs can entrap circulating cancer cells and, in that way, facilitate metastasis. A better understanding of the crosstalk between cancer and NETs can help to devise novel approaches to the therapeutic interventions that block cancer evasion mechanisms and prevent metastatic spread.

**Abstract:**

The present review highlights the complex interactions between cancer and neutrophil extracellular traps (NETs). Neutrophils constitute the first line of defense against foreign invaders using major effector mechanisms: phagocytosis, degranulation, and NETs formation. NETs are composed from decondensed nuclear or mitochondrial DNA decorated with proteases and various inflammatory mediators. Although NETs play a crucial role in defense against systemic infections, they also participate in non-infectious conditions, such as inflammation, autoimmune disorders, and cancer. Cancer cells recruit neutrophils (tumor-associated neutrophils, TANs), releasing NETs to the tumor microenvironment. NETs were found in various samples of human and animal tumors, such as pancreatic, breast, liver, and gastric cancers and around metastatic tumors. The role of the NETs in tumor development increasingly includes cancer immunoediting and interactions between the immune system and cancer cells. According to the accumulated evidence, NETs awake dormant cancer cells, causing tumor relapse, as well as its unconstrained growth and spread. NETs play a key regulatory role in the tumor microenvironment, such as the development of distant metastases through the secretion of proteases, i.e., matrix metalloproteinases and proinflammatory cytokines. NETs, furthermore, directly exacerbate tumor aggressiveness by enhancing cancer migration and invasion capacity. The collected evidence also states that through the induction of the high-mobility group box 1, NETs induce the epithelial to mesenchymal transition in tumor cells and, thereby, potentiate their invasiveness. NET proteinases can also degrade the extracellular matrix, promoting cancer cell extravasation. Moreover, NETs can entrap circulating cancer cells and, in that way, facilitate metastasis. NETs directly trigger tumor cell proliferation through their proteases or activating signals. This review focused on the pro-tumorigenic action of NETs, in spite of its potential to also exhibit an antitumor effect. NET components, such as myeloperoxidase or histones, have been shown to directly kill cancer cells. A better understanding of the crosstalk between cancer and NETs can help to devise novel approaches to the therapeutic interventions that block cancer evasion mechanisms and prevent metastatic spread. This review sought to provide the most recent knowledge on the crosstalk between NETs and cancer, and bring more profound ideas for future scientists exploring this field.

## 1. Neutrophils and NETs

Polymorphonuclear neutrophils (PMNs), the most abundant white blood cells, are frontline fighters against invading microorganisms. PMNs destroys pathogens, or other endogenous or exogenous factors, using a combination of mechanisms, including phagocytosis, oxidative bursts, the release of antimicrobial mediators, and the production of neutrophil extracellular traps (NETs) [1]. NETs are web-like structures built from nuclear or mitochondrial DNA fibers, decorated with anti-microbial enzymes and histones, which are released to entrap and kill pathogens [2]. Besides their role as an anti-microbial weapon, NETs create a physical barrier for both pathogens and immune cells. The process of NET formation in its classical form is called NETosis and has been defined as a type of regulated cell death distinguished from apoptosis and necrosis [3]. Further studies have described an alternative pathway of a non-cell-death NETs generation, named vital NETosis. NET release is initiated by an oxidative burst via raf-MEK-ERK activation of NADPH oxidase. Subsequently, neutrophil elastase (NE) translocates from azurophil granules into the nucleus, where it instigates chromatin breakdown through histone hydrolysis. Further observations have suggested that myeloperoxidase (MPO) has also been implicated in chromatin decondensation and the rupturing of the nuclear envelope. Chromatin decompaction is further supported by peptidyl arginine deiminase 4 (PAD4)—a protein-citrullinating enzyme that enters the nucleus to deiminate specific arginine residues on histones, resulting in the loss of positive charge from the transformed arginine residues and the disassembling of nucleosome structure [4]. Crucial steps in NET formation include nuclear swelling, nuclear envelope disintegration, the mixing of nucleic acids and granule proteins within a large intracellular vacuole, the spilling of nuclear content into the cytoplasm, and, finally, cell membrane breakdown [5].

## 2. NETs—Friend or Foe?

NETs not only act as a host defense mechanism, but also play a pivotal role in infectious and non-infectious conditions [5,6,7]. While the beneficial effects of NETs in fighting pathogens have already been largely described, the detrimental role of NETs is rapidly beginning to emerge. Netting neutrophils play a significant role in the pathogenesis of various diseases, such as systemic lupus erythematosus, small vessel vasculitis, rheumatoid arthritis, preeclampsia, cystic fibrosis, psoriasis, and, as recently described, in Covid-19 [5,6,7]. NET generation and degradation in patients with granulomatosis with polyangiitis and systemic lupus erythematosus is impaired [6,7]. NETs are also implicated in various other pathological processes, such as coagulation disorders, diabetes, atherosclerosis, wound healing, and periodontitis [8].

### 2.1. Heterogeneity of Neutrophils

Although neutrophils have long been considered as a terminally differentiated, homogenous cell population of the innate immune response, different studies started to highlight the heterogeneity of their phenotypes and the versatility of their functions [5]. The phenotype and function of a resident neutrophil is the result of a specific maturation program and/or inflammatory signals from surrounding milieu (cytokines, chemokines, enzymes, growth factors, various lipids and proteins) translating various environmental signals into specific gene and transcription factor programs. This paradigm is supported by the presence of distinct neutrophil precursors at different stages of development contributing to the diversity of mature neutrophils [9].

### 2.2. Tumor-Infiltrating Neutrophils

The tumor microenvironment (TME) comprises different non-malignant cell types and an extracellular matrix (ECM), altogether named the stroma. The stroma consists of the basement membrane, immune cells, cancer-associated fibroblasts (CAFs), pericytes, and vascular endothelial cells [10]. Tumor cell proliferation, the evasion of immune surveillance, and the spread and metastasis are affected by the changes in the composition, function, and communication between all stromal components [10,11]. Amongst various immune cells within the TME, such as dendritic cells, lymphocytes, macrophages, granulocytes, and fibroblasts, infiltrating neutrophils, in concert with other cell types, play a prominent role in cancer development [11]. However, the pro-tumor functions of tumor-infiltrating neutrophils have only recently come to the light. Consistently, various mediators produced by tumor or stromal cells stimulate granulopoiesis, neutrophil release from the bone marrow, and the migration of these cells [11]. These mediators include growth factors: G-CSF, GM-CSF and CXC chemokines, and CCL3 [11]. Recently, different studies started to highlight that cancer cells release chemokines attracting neutrophils to tumor microenvironments [12,13]. In the recent past, tumor-associated neutrophils (TANs) have emerged as important contributors to the tumor biology. However, consistent and continuous evidence has confirmed that these cells appear to play an important role in the entire process of cancerogenesis, followed by the metastatic spread to distant organs [12]. TANs are capable of polarization into two populations (N1 and N2) according to cytokine production patterns and effector functions. These two populations present either an anti-tumorigenic “N1” phenotype or, fed by TGFβ, a pro-tumorigenic “N2” phenotype [12]. Both N1 and N2 cells bear similar surface markers to peripheral blood neutrophils, i.e., CD66b+, CD11b+, CD15+, CD16+, HLA-DR−, and arginase-1+ [13]. In fact, due to the often-shared cell morphology and the overlap of the expression of these surface markers between the different functional groups, it is difficult to clearly distinguish between the subtypes N1 and N2 [13]. N1 neutrophils can effectively eliminate tumor cells via lysis, indirect cytotoxicity or the induction of tumor cell apoptosis. N1 cells exhibit increased cytotoxicity and a reduced immunosuppressive ability due to the increased release of TNFα, Fas, ICAM-1, and ROS, and through a decreased arginase expression [14]. On the other hand, N2 cells promote immunosuppression, support tumor growth, invasion, epithelial–mesenchymal transition (EMT), angiogenesis and the metastasis of cancer cells [15]. N2 neutrophils express high levels of arginase, MMP-9 VEGF, and numerous chemokines (for example CXCL4, CCL2, and CCL5). The affluence of these cells corresponds with poor clinical outcomes [15]. The tumor-secreted TGF-β was shown to transform N1 TANs (tumor-suppressive phenotype) into N2 TANs (tumor-promoting phenotype) [15]. Infiltrating neutrophils continue to promote tumor development by secreting pro-inflammatory and pro-angiogenic chemokines and cytokines, such as matrix metallopeptidase 9 (MMP9) and interleukin 6 (IL-6) [14,15]. Circulating tumor cells shed from the primary tumor sites are disseminated via blood or lymphatic vessels and reach distant organs. In a recent study, neutrophils emerged as important players supporting circulating tumor cells survival during hematogenous dissemination [16]. Furthermore, it was confirmed that neutrophils escort circulating tumor cells, increasing the dynamics of cell cycle progression [16]. Wculek et al. have identified neutrophils as the main drivers in establishing the pre-metastatic microenvironment in different murine breast cancer models [17].

## 3. NETs Are Present in Tumor Microenvironment

The discovery of NETs has created a completely new field of investigation in oncology. The first evidence of NET formation by tumor-associated neutrophils in human tissues came from a histopathological analysis of diagnostic biopsies from Ewing sarcoma. Out of eight tissue samples, TANs were found in six specimens and NETs in two patients. In this study, NET formation was associated with relapse and metastatic disease, despite chemotherapy treatment [18]. Several further studies revealed the presence of NETs in peripheral blood and tumor specimens from animals and cancer patients. NETs were found in tumor samples from primary and metastatic sites. Murine neutrophils from animals with leukemia, mammary, and lung cancer were more prone to release NETs compared to granulocytes of healthy mice. Overproduction of NETs went in parallel with activation of intravascular coagulation and the presence microvascular thrombosis in these animals [19,20]. Currently, there are scarce published data regarding the occurrence of NETs in clinical samples from patients with hematological malignancies [21,22]. Nie et al. reported that neutrophils are prone to produce NETs in hematological malignancies, such as chronic lymphocytic leukemia, and participate in disease progression via TLR9 signaling [21]. Cedervall et al. discovered that the number of netting neutrophils in the kidneys and hearts of tumor-bearing animals (MMTV-PyMT—breast cancer and RIP1-Tag2—insulinoma) is increased. The kidney involvement in these animals is accompanied with concomitant kidney insufficiency. DNase (NET-degrading enzyme) treatment recovered renal function in experimental animals, pointing to the pathogenic role of NETs in acute kidney damage [23]. In another in vitro study, it was demonstrated that extracellular RNAs from Lewis lung carcinoma cells induced the release of NETs [24]. TANs were active in the low-oxygen environment with the presence of proinflammatory cytokines, such as IL-8, IL-1β, and G-CSF [25,26]. The molecular mechanism of NET formation in TME is also dependent on the nuclear factor high mobility group box 1 (HMGB1), which, by binding to TLR4, induce activation of p38 MAPK/ERK signaling pathways, further contributing to the excessive release of inflammatory cytokines [27].

## 4. Circulating NET Markers in Cancer Patients

The plasma NET markers include citrullinated histones (H3Cit-DNA), cell-free DNA (cfDNA), neutrophil elastase (NE), and nucleosomes [28]. All circulating markers can be easily measured in human plasma. NET marker concentrations in the plasma of different cancer patients, including lung, pancreatic, and bladder cancer, were found to be higher than in healthy controls [29]. In lung cancer patients, Li et al. demonstrated the presence of NETs in lung tissues, peripheral blood, and sputum [24]. The circulating levels of NETs (DNA-histone complex, double-stranded DNA, NE) were measured in the peripheral blood of liver cancer patients, along with contact system activation markers. Both NETs and contact system activation markers were higher in cancer patients than in healthy volunteers [30]. In accord with the above-mentioned observations, Rosell et al. confirmed the presence of circulating markers of neutrophil activation and NET formation (NE, H3Cit-DNA) in 106 patients with terminal cancer with concomitant hypercoagulation and hyperfibrinolysis. They found that NET markers had a prognostic value in terminal cancer patients. NE and H3Cit-DNA were both associated with a poor clinical outcome. Interestingly, although the markers of coagulation and fibrinolysis were elevated, they did not have a prognostic significance in the patients of this study. Moreover, the correlations between NETs and coagulation/fibrinolysis markers were weak or non-existing. This observation suggests that NETs contribute to poor prognosis in terminal cancer through mechanisms independent of thrombosis [31]. Consistently Oklu et al. [29] detected high levels of nucleosomes, cfDNA, DNase-1, the thrombin-antithrombin III (TAT) complex, as well as endonuclease-G and its activity in plasma from cancer patients. Additionally, NETs were found and quantified by fluorescent immunohistochemistry in tumor tissue samples and venous thrombi of cancer patients. These authors have found that plasma samples from cancer patients contained higher levels of nucleosomes and free-circulating DNA compared to the non-cancer group. A Western blot analysis revealed a significantly lower level of DNase-1 protein that paralleled a lower nuclease activity in plasma samples from cancer patients compared to non-cancer subjects. Venous thrombi from cancer patients and tumor tissue from liver and lung cancer also showed increased presence of NETs. However, high levels of NETs in cancer patients did not correlate with TAT complex activation or the incidence of venous thrombosis in these patients [29]. The objectively measured diagnostic, prognostic, and predictive biomarkers of tumors are desperately needed in clinical practice. The assays quantifying the circulating NET markers should be developed into commercially available laboratory tests validated in human plasma samples that are easily accessible. This will allow for the potential clinical implementation of such tests as prognostic tools, or as guides to the decision-making process necessary in cancer therapy.

## 5. NETs Fuel Cancer Progression and Indicate Poor Prognosis

Theoretically, NETs might have potential anti-tumorigenic effects through the direct killing of cancer cells or the activation of the immune system. Like histones, NE, and MPO in vitro, NET components destroy tumor cells and block tumor growth and metastasis formation [32,33,34]. Surprisingly, accumulating evidence suggests that NETs exert multifaceted protumorigenic effects. Different studies have highlighted the prominent role of this structure in the progression and enhancement of metastatic potential of animal and human tumors. Richardson et al. confirmed the association between in vitro NET release by stimulated neutrophils and the poor prognosis in colorectal cancer patients [35]. The role of NETs in tumor immuno-editing has been investigated in the previously mentioned pediatric Ewing sarcoma study of Berger-Achituv et al. [18]. These authors demonstrated the presence of NETs in tissue samples of Ewing sarcoma pediatric patients with an early relapse after high doses of chemotherapy, suggesting a possible role of NETs in Ewing sarcoma progression [18]. Similarly, in histopathological specimens of colorectal liver metastases from patients who underwent selective curative resection, Tohme et al. found an abundance of TANs and NETs in comparison to normal liver tissue [36]. Citrullinated histones were also differently expressed in tumor samples compared to normal tissue. Furthermore, preoperative levels of MPO-DNA, a well-known marker for systemic NET release, were higher in patient serum than in healthy controls and were associated with poor disease-free survival and overall survival. Thus, MPO-DNA serum levels could represent a possible prognostic biomarker in these patients [36,37]. Kanamaru et al. [38] found that CD66-positive mature light-density neutrophils (a subpopulation of neutrophils with enhanced capability of producing NETs) were clustering in the peritoneal cavity of patients who underwent laparotomy due to gastric cancer. NET presence was found to be related to abdominal recurrence of cancer [38]. Moreover, neutrophils from cancer patients showed a higher amount of H3Cit than normal cells. Additionally, higher levels of plasma H3Cit were observed in more advanced stages of cancer [12]. This study confirmed that light-density neutrophils play a critical role in tumor invasiveness [12]. Surprisingly, H3Cit in the plasma of cancer patients did correlate with activators or products of NETs, such as MPO, NE, IL-8, and IL-6 [39]. Interactions of NETs with coagulation systems have become increasingly apparent in cancer. NETs induce the intravascular activation of the blood clotting cascade (cancer-associated thrombosis) that contributes to primary tumor growth, cancer aggressiveness, progression, and metastasis [40]. According to Lima et al. [40], there is a significant correlation between the incidence of thromboembolic events and a worse prognosis of neoplastic disease. These authors suggested that the NETs assembled on a scaffold with thrombus and may play an important role in cancer pathogenesis in concert with the hemostatic system [40]. A large body of evidence has indicated that both circulating NET-derived and hemostatic factors play a key role in tumor development, such as the angiogenesis, metastasis, and modulation of innate immune responses [40]. Consistently, Jung and al. showed that NETs stimulate cancer-associated thrombosis correlated with a worse outcome [41]. It is well known that the incidence of thromboembolic disease markedly depends on cancer type. For instance, patients with breast cancer have a low rate of thromboembolic events, whereas patients with pancreatic cancer have a high rate [42]. Pancreatic cancer patients are at high risk of developing venous thrombosis attributed to NET production, as confirmed in an orthotopic cancer model in mice and patients [43].

### 5.1. How Do NETs Awaken Dormant Cancer Cells?

Cancer cells from a primary tumor can migrate to other tissues, remaining dormant and clinically silent for a long time. The concept of tumor cell dormancy has been described for most common solid cancers, including breast, prostate, lung, colon, and kidney cancers, as well as melanoma. Hematological malignancies, such as multiple myeloma, lymphoma, and leukemia, were included as well [44]. The slow-cycling cancer cells can disseminate early and seed secondary organs where they wait to be awakened, thus causing cancer to recur. Dormant cancer cells settle in specific niches. For example, breast cancer cells inhabit the perivascular regions of the lung [44]. The exact mechanisms causing the awakening, the restart of proliferation, and the metastasis of the slow-cycling cells overlooked by the immune system (immune evasion) are largely unknown. It has been reported that NETs possess the ability to wake dormant cancer cells, and are thus responsible for tumor relapse and metastatic spread [45]. Consistently, NETs formed in the course of the inflammatory process have awakened malignant cells in experimental tumor models. In an excellent study Albrengues et al. proved that NETs released in the course of chronic pulmonary inflammation awaken dormant breast cancer cells and promote metastatic spread [45]. The chronic lung inflammation in this model was induced by infection or cigarette smoke. Using a cell cycle reporter to measure dormancy against the reactivation of cancer cells, these authors found that prolonged inflammation induced by repeated lipopolysaccharide (LPS) inhalation caused dormant cancer cells to restart proliferation, and this process was dependent on the presence of intact neutrophils. The dormant malignant cells could be awakened by LPS even a month after they had inhabited the lungs. The effect of NETs on the cancer was exerted indirectly via extracellular matrix (ECM) remodeling. The analysis revealed that NET proteinases, NE and MMP9, cleaved laminin, revealing new epitopes of this molecule. Such modified laminin activated integrin α3β1, which in turn re-initiated cancer cell proliferation. The researchers confirmed the presence of cycling cells close to remodeled laminin, and on the contrary, the cells near intact laminin remained dormant. Blocking the new epitope of laminin with dedicated antibodies hindered the awakening of cancer cells, both in vitro and in vivo. Furthermore PAD 4 inhibitor or DNAase treatment impeded the formation of NETs and prevented the activation of quiescent cells and metastasis formation [45]. Recent discoveries have suggested that NE and MMP9 blockades in vitro prevent cancer from re-entering cell cycle and block LPS-mediated cancer progression in vivo. Furthermore, inhibiting NET formation further prevented neutrophil accumulation, thus breaking the vicious cycle of self-perpetuating inflammation. These effects were also reported by studies of Orgaz et al., who revealed that NET proteases, such as MMP9, are associated with metastatic dissemination [46]. Continuing this experimental work, Albrengues et al. found that not only laminin, but also thrombospondin-1 (TSP-1), was disintegrated by NE and MMP9 [45]. TSP-1 upregulates integrin 6 subunit expression, thus promoting tumor cell adhesion to laminin, and subsequently supporting malignant cell invasion [47]. The observations of Albrengues et al. suggest that TSP-1 abolished the effect exerted by cleaved laminin-111 on cell proliferation, thus, TSP-1 prevented metastatic relapse by proteolytic remodelling of laminin-111 [45]. Albrengues et al. thus concluded that both TSP-1 degradation and laminin remodeling are necessary to awake quiescent cells in their niches. Integrin β1 accounts for the activation of FAK-ERK-MLC2-YAP signaling pathway, contributing to proliferation and survival of malignant cells. In accord with this observation, NET-induced activation of the same pathway, requiring NE and MMP9 activity, awakes slow-cycling cancer cells. Whatever the precise mechanistic basis of this process may be, experiments with RNAi silencing suggest that α3β1 integrin and transcriptional regulator YAP in cancer cells are necessary for NET-dependent awakening of dormant cancer cells. The study of Albrengues et al. confirmed the hypothesis of “seed and soil”, i.e., the predilection for metastasis to specific organs where the local microenvironment is favorable [45,48]. Amongst the many components of the tumor microenvironment (soil), neutrophils, and their products, all play a prominent role in tumor (seeds) progression, the evasion of the immune system, and metastasis [45].

### 5.2. How NETs Promote Cancer Invasion, Evasion, Its Spread, and Metastasis Formation

**Figure 1:**
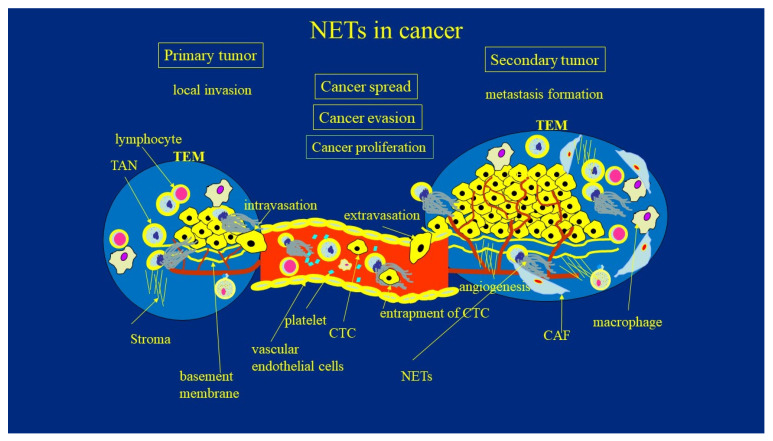
The role of NETs in cancer development. TAN—tumor associate neutrophils; TEM—tumor environment; CAF—cancer associated fibroblasts; NETs—neutrophil extracellular traps; CTC—circulating tumor cells.

The systemic spread and formation of metastases in distant organs is responsible for the majority of cancer deaths. A multi-step process of metastasis formation includes local invasion, intravasation, and the survival of tumor cells in the circulation, which is followed by extravasation from blood or lymphatic vessels, the colonization of distant sites, the awakening from dormancy, and the metastatic spread. At each step of this complex process, malignant cells must also resist attacks from the host’s immune system. There is experimental evidence suggesting that NETs participate at every stage of this process, given their versatile role in the metastatic cascade. (Figure 1).

### 5.3. NETs Supports the Cancer Evasion Strategies

The evasion of tumor cells from immunosurveillance depends on the interplay between various infiltrating immune cells and tumor cells. Whatever the mechanistic basis, it appears that immunosurveillance of tumors is canonically dependent on the presence of the major histocompatibility complex 1 (MHC1) antigens on cancer cells enabling lymphocytes T (both CD4+ and CD8+) to discriminate tumor cells from normal cells, as well as to control the tumor cell survival [49,50]. Various proteolytic enzymes (proteinases) are able to modulate the cell-surface-associated presentation of MHC molecules. MMP9, for example, is responsible for the shedding of MHC class I antigen from cancer cells [51]. The selected subclones of malignant cells achieve the capability to hide from the immune system by losing the ability to present cancer antigens to T-cells. Various components of ECM have been recognized as sources of signals for the immune system to slow down immune reactions, for example through the expression of checkpoint molecules. The ECM is a reservoir of immunomodulatory cytokines and growth factors that are released upon their proteolytic degradation. Metalloproteinases and NE can modulate immune and inflammatory responses through the degradation of the ECM. The cleavage products of the ECM (e.g., matrikines) can, by themselves, affect immune surveillance. NET proteases can impede the immune response and, thus, ensure the best possibility of cancer cell survival by enabling the metastatic process [45]. According to Albrengues et al. [45], the degradation of matrix proteins is one of the mechanisms of tumor evasion that silences the host’s immune system. NET proteinases stimulate the production of IL-8, IL-1β, and TNF-α with tumor-associated macrophages through the activation of several MMPs. This process is dependent on Src kinase activation, highlighting the fact that NE also impacts integrins and integrin-mediated intracellular signaling [22,52]. As another example, the inhibition of hyaluronic acid (a major component of the ECM) synthesis by 4-methylumbelliferone in a mesothelioma xenograft has led to a significant increase in the expression of both immune checkpoint molecules, PD-1 and PD-L1 [53]. Although the mechanisms involved in ECM modification by NET components are not fully elucidated, the clear connection between the ECM composition and proteinases, as well as the immune escape, strongly support the existence of such an effect. Onuma et al. [54] confirmed that the blockade of NETs, in combination with immune checkpoint PD-1 inhibition, improved the response rates of colorectal cancer metastases to immune checkpoint inhibitors as a single therapy. This was achieved through the improving of the function of exhausted CD8+ T-cells [54].

### 5.4. NETs Enhance Invasion Capacity of Cancer Cells

A crucial event at the first stage of metastatic colonization is the formation of a favorable niche for tumor engraftment attributable to tumor–stroma crosstalk [55]. The process of metastatic spread began from proteolytic remodeling of ECM and the release of ECM metabolites necessary, or even mandatory, for the dissemination of cancer cells. As mentioned above, the ECM is digested by MMPs, disintegrin, metalloproteinases with thrombospondin motifs (ADAMTS), and proteases that specifically cleave at cysteine, serine, and threonine residues [56]. Several components of mature NETs cause an imbalance in the microenvironments, as well as the emergence of metastatic niches. For example, NET-derived NE and MMP-9 degraded ECM to actively induce tumor invasion [57]. Accordingly, it was shown that matrix metalloproteinase catalytic activity modulated the invasiveness and provided a route for the malignant cells to metastasize via modulation of the integrins–FAK signaling pathway [58]. In an experimental model using Boyden transwell invasion assay, Park et al. [59] focused on the neutrophil-mediated invasion of tumor cells. The applied model confirmed that tumor invasion through the filter in the transwell system can be promoted by the mutual interaction between tumor cells in the upper chamber and neutrophils in the lower chamber. Furthermore, the blockade of NE and matrix metalloproteinases impeded tumor invasion [59]. In agreement with this observation, DNase I treatment downregulated NE and NET activities and reduced the invasive and metastatic potential of malignant cells [59]. Other investigators were able to confirm significant correlation between NETs and liver metastases of patients with breast and colon cancers, thus confirming increased binding activity of transmembrane protein CCDC25 on primary cancer cells to NET DNA. These authors proved that CCDC25 senses extracellular DNA and, subsequently, activates the ILK-β-parvin pathway to attract cancer cells. NET-mediated metastasis was abrogated in CCDC25-knockout cells. Moreover, the expression of CCDC25 was associated with a poor outcome of the disease [60]. Although the detailed mechanism of tumor invasion and metastasis via NET molecules is still not completely understood, it would be interesting to investigate the role of TANs in the regulation of NET-mediated tumor invasion. Signaling is an integral process in controlling invasive and metastatic potential of tumor cells. The signaling between various structures in TME, including NETs fragments, is crucial in controlling the invasive potential of the tumor. Thus, in silico studies modelling these critical interactions and their effects are warranted to discern alternative explanations of these processes and pave the way for the development of new therapeutic strategies [14].

### 5.5. NETs Enhance Systemic Spread and Tumor-Associated Angiogenesis

Tumor cells can migrate and intravasate the blood or lymph vasculature. They can survive within the circulation, then extravasate at distant sites. The factors determining adhesion strength, which might influence the ability of cells to transmigrate through an endothelial cell monolayer and the basement membrane, are poorly understood. Recent studies have highlighted that these processes are driven not only by signals from cancer cells, but are also modified by signals from components of the TME [55]. Current evidence suggests that NETs may play a crucial role in the hematogenous spread of tumors. Jung et al. [41] showed that NETs promoted tumor growth, metastasis, and angiogenesis of the pancreatic cancer cell line (AsPC-1). NETs used as chemoattractans stimulated AsPC-1 cell migration (in a Matrigel-coated invasion chamber) better then intact neutrophils. These effects were abrogated by histone-binding agents (heparin, polysialic acid), DNAse I, and Toll-like receptor neutralizing antibodies. Antibodies against both TLR2 and TLR4 significantly inhibited NET-mediated AsPC-1 cell migration. Although not unexpectedly, these results support the opinion that TLR2 and TLR4 participate in tumor transmigration. In patients with pancreatobiliary malignancy, elevated NET markers correlated with hypercoagulability makers. Histone–DNA complexes were used as markers of NETs. Another component of NETs, histones, significantly increased the endothelial cell proliferation and the formation of new blood vessels in a dose-dependent manner. Application of histone-binding agents abrogated histone-induced angiogenesis [41]. The same directionality of the effect was observed by Tohme et al., who reported that the chemotactic factor released during NET formation may stimulate proliferation and migration of cancer cells [36]. Finally, the transmigration mechanisms were explained by Kołaczkowska et al., who observed the adherence of circulating NETs to blood, resulting in increased cancer extravasation efficiency, which would enable cancer cells to cross the endothelial barrier [61]. On the other hand, the previously mentioned report of Park et al. suggested that not DNA itself, but rather NET-related proteases are responsible for this effect [59]. Such a discrepancy may be explained by the fact that such a structure as complex as the one between NETs and the locally concentrated enzymes, must be taken as an inseparable assembly, rather than a conglomerate of individual components. Once in the circulation, tumor cells become entrapped by NETs DNA threads. Through the use of cecal ligation, Cools-Lartigue et al. [62] demonstrated the presence of circulating lung carcinoma cells wrapped in NET DNA conglomerates in a murine model of infection. Consequently, circulating “packages” were seeded in the liver, forming micrometastases within 48 h and secondary liver cancer 2 weeks after the cancer cell injection. DNAse or NE inhibitors abrogated the effects [62]. Evidence consistent with these observations was provided by Najmeh et al. from the same group, who found a significant association between upregulation of β1-integrin and NET-related entrapment of circulating lung carcinoma cells, further facilitating metastasis formation and cancer spread [63]. Whatever the precise basis of this mechanism is, it appears that inflammatory mediators harbored by neutrophils may be responsible for insufficient clearance of circulating cells [64]. NETs’ entrapping abilities can be, at least partially, attributed to the ability to adhere to DNA mesh carried by the variety of integrins expressed on the surface of cancer cells. Such interaction was completely abrogated by DNase 1 [65]. Furthermore, the TAN-CTC adhesion process facilitates cancer cell extravasation through the breaking of the transendothelial barrier [66]. The proposed adhesive interaction between circulating neoplastic cells and TANs leads to the increased endothelial cell contraction, permeability, and malignant cell extravasation [66]. A multi-level model shed new light on the fundamental processes elucidating the role of NETs in cancer invasions, transport, and transendothelial migration, thus taking into account specific NET–cell adhesion, ECM–tumor–NET interaction, and intracellular signaling [67,68,69,70,71]. Further studies, however, are still warranted to explore these issues.

### 5.6. How NETs and Tumor Communicate

The interaction between the tumor and NETs is reciprocal. In their excellent paper, Demeters et al. compiled initial reports showing that TANs are a potent source of NETs and, on the other hand, cancer cells can stimulate neutrophils to release NETs as shown in various animal models of cancer [19]. NETs enhance the gathering and proliferation of single cancer cells, contributing to tumor metastasis by releasing MMP and NE, which through the degradation of ECM, paves a way for tumor cells to leave the primary niche and to migrate to other organs. Conversely, inflammatory cytokines, such as IL-8 and granulocyte colony-stimulating factor, as well as various soluble factors, i.e., exosomes released from cancer cells, stimulate neutrophils to release NETs [72]. Metastatic cancer cells possess the ability to stimulate the release of NETs directly and without the engagement of inflammatory mechanisms [33]. According to the model of a vicious circle proposed by Park et al., the metastatic breast cancer cells induced neutrophils to form NETs, which further enhanced tumor cell growth in target organs [59]. McInturff et al. demonstrated that cancer cells themselves are able to stimulate neutrophils to form NETs in a hypoxic environment where solid tumor growth is enhanced by the higher expression of HIF-1α [73]. Another mechanism by which cancer cells may stimulate neutrophils to form NETs depends on the production of IL-8 and the release of exosomes which require additional priming with granulocyte colony-stimulating factors. Leal et al. found that tumor-derived exosomes of cancer patients in a hypercoagulable state can induce NET release, and that NETs can serve as a scaffold for coagulation factors, platelets, and exosomes carrying prothrombotic mediators, altogether promoting the development of thrombo-embolic complications and cancer progression [72]. In an excellent review, Yousefi et al. summarized various experimental evidence that lung, colon, ovarian, and anaplastic thyroid cancer (ATC) cells induce the release of mitochondrial extracellular DNA traps by viable neutrophils [74]. Furthermore, tumor cells have been demonstrated to produce IL-8, attracting myeloid-derived suppressor cells and activating neutrophil precursors to release NETs [75]. Similarly, liver ischemia reperfusion in a murine model resulted in NET extrusion in parallel with the progression of metastatic disease, while the pre-treatment of mice with topical DNase or a PAD4 inhibitor abrogated these effects [36]. Consistently with these observations in mice, an increased postoperative NET formation inversely correlated with the disease-free survival in patients undergoing liver resection for metastatic colorectal cancer [36]. However, the limitation of this study manifested in the use of NET plasma markers (MPO–DNA complexes) as surrogates of netting capacities of neutrophils rather than a direct analysis of NET presence in the examined tissues.

### 5.7. NETs in the Formation of Metastatic Niche

Tumors metastasize to distant organs with tissue-specific microenvironments, which are very different from that of a primary tumor. The precondition of distinct microenvironments involving ECM remodeling and the creation of a favorable pre-metastatic niche is necessary for the seeding of new tumor colonies [56]. The most common modification of the ECM in the primary TME is increased collagen deposition. On the contrary, fibronectin dominates along with glycoproteins and proteoglycans such as tenascin C, osteopontin, and versican in a pre-metastatic niche [76]. The primary niche is mainly formatted by mediators released by growing tumor cells, further acting on various components of TME, which in turn release a second generation of molecules, directly creating a favorable microenvironment. NETs participate in this process, conferring the effect on the electrostatic charge and conformation of fibronectin and collagens in the process of citrullination. This effect is mediated by the enzyme PAD4, derived from NETs during pre-metastatic niche formation [23,77]. Moreover, NETs equipped with proteases are highly associated with aggressive tumor growth and invasion, but this high metastatic potential is abrogated by DNase I treatment [14,59]. Recently, different studies began to highlight that epithelial–mesenchymal transition (EMT), a process by which epithelial cells acquire mesenchymal properties endowing cancer cells with invasive and metastatic potential, is driven by NETs [78,79]. Martins-Cardoso et al. recently described the association between NETs and the pro-metastatic phenotype of human breast cancer cells [78]. Co-cultures of tumor cells treated with isolated NETs underwent several experiments, including migration assay, quantitative RT-PCR, Western blotting, immunofluorescence, and flow cytometry assays. RNA-seq data from The Cancer Genome Atlas (TCGA) database were also assessed [79]. NET components changed the epithelial into mesenchymal phenotype (upregulated expression of N-cadherin and fibronectin, and downregulation of E-cadherin). The effect was accompanied by the increased motility of cells. RNA-seq revealed pro-inflammatory and pro-metastatic signatures. Accordingly, TCGA data analysis of samples from breast cancer patients showed a significant correlation between neutrophil and the pro-tumoral signature of gene expression [78]. Further studies have shed light on the crosstalk between glioma progression and NETs in TME. The tumor growth was mediated via the HMGB1/RAGE/IL-8 axis [80]. Covid-era discoveries also led to the conclusion that lung inflammation and a cytokine storm accompanied by NET formation in the course of COVID-19 contributes to dormant cancer cells awakening and the formation of a pro-metastatic niche [81].

### 5.8. NETs Is Physically Blocking T-Cell Infiltration to the TME

It has been demonstrated that PMNs and their products are engaged in multiple interactions with T-lymphocytes and a molecular basis of these associations is being explored. A number of important links between NETs and functions of T-lymphocytes have been discovered [82,83,84]. The communication between T-cells and NETs occurs either via direct contact of lymphocytes with the NET backbone or depends on released mediators, including enzymes, cytokines, and radical oxygen species. Tillack et al. [83] showed that NETs can directly reduce the T-cell activation threshold in response to specific stimuli. Both NET/cell contact and TCR signaling are necessary for T-cell priming [83]. Bilyy et al. demonstrated that NETs form a barrier between necrotic and viable areas in acute abdominal inflammation [84]. In a very recent study, Surashri Shinde-Jadhav et al. [85] discovered a direct link between intratumoral NETs and T-cell cytotoxicity. These authors demonstrated that NETs formed a barrier between the irradiated tumor and stroma, blocking the invasion of CD8 T-cells to TME [85]. NETs were found to be surrounding CD8 T-cells but not colocalizing with them. Moreover, increased intratumoral CD8 T-cell infiltration was noted in tumors of mice treated with DNAse I [85]. These authors claimed that NETs may play a role in tumor radioresistance by blocking intratumoral CD8 T-cell infiltration post-RT. This effect was related to the clinical effect of the RT [85]. A higher intratumoral PMN to CD8 ratio was observed in RT non-responders compared to RT responders. Additionally, these authors found that patients with persistent disease had a high pre-treatment intratumoral PMN to CD8 ratio. In line with this experimental data is the clinical observation that a high PMN to CD8 ratio was associated with worse overall survival [85]. The NET barrier at the interface of tumor cells and necrotic tissue was also noted by the previously mentioned study of Berger-Achituv et al. in Ewing sarcoma biopsy samples [18]. These authors proposed, for the first time, that this NET barrier may enable tumor immune escape [18]. These observations are in accord with the report of Teijeira et al., who show that intratumoral NETs block the contact between tumor cells and cytotoxic cells [86]. Summarizing, NETs provide a physical barrier protecting from the spread of infectious agents, thus localizing the infection. On the other hand, this mechanism is not beneficial in the course of tumor development as it contributes to the immune evasion mechanisms by blocking the access of cytotoxic cells to the growing tumor.

## 6. Potential Anti-NETs Therapy of Cancer

Targeting NETs is a relatively new option with significant potential for the treatment of PMN-mediated disorders. This review focused on the pro-tumorigenic activity of NETs, highlighting their ability to serve as an appealing therapeutic target for cancer. An interesting option is also the combination of anti-cancer and anti-NET intervention. With the recent advances in the knowledge of how NETs are generated or how to dismantle their structure, several approaches can be considered to develop strategies to prevent the awakening of dormant cancer cells and to inhibit the spreading of tumors, as well as the formation of metastases. The detection of NETs in tumor biopsies or the presence of NET markers in the circulation may stand for the most accurate method of identifying patients who could benefit from NET-targeting therapy. Recently, a number of different researchers have presented emerging and promising concepts for cancer treatment based on the anti-NETs strategy. Park et al. showed that inhibiting NET formation or dismantling NETs with DNase I-coated nanoparticles markedly reduced lung metastases of breast cancer in mice [59]. The effectiveness of such an approach was confirmed by the experimental evidence of the inhibitory effect of DNase-I on the invasion and migration of breast cancer cells in vitro concomitantly with NET degradation [59]. Other groups of scientists have stated that cannabinoids, which act through the cannabinoid receptor, suppress PMN functions, including cell migration, production of ROS, and TNF-α production followed by NET release [87,88]. Very recently, Munir et al. reported that cancer-associated fibroblasts secreted amyloid β, modulating tumor-associated NET release through CD11b in a ROS-dependent manner [89]. This effect was observed both within the TME and at systemic levels in the blood and bone marrow. The prevention of amyloid β release abrogated tumor growth and restored an anti-tumor status in TANs, suggesting a potential therapeutic strategy on various cancer types [89]. In the previously discussed paper, Albrengues et al. [45] provided compelling evidence that antibodies against NET-remodeled laminin prevented awakening of dormant cells. These results provide a rationale for targeting this pathway to treat metastatic cancer and to prevent the disease relapse [45]. A comprehensive analysis of the signaling pathways regulating PAD4 activation may result in the generation of pharmaceuticals that target NET-related disorders. Such a strategy could be applied to prevent chromatin decondensation and the expulsion of chromosomal DNA and, what is more, to decrease metastatic behavior of cancer. A large panel of available PAD4 selective inhibitors have been developed recently [90]. It was demonstrated that PAD4 knockout inhibited tumor growth and metastasis of colorectal cancer by preventing the citrullination of the ECM in the liver and impeding the subsequent epithelial-to-mesenchymal transition [90]. Moreover, inhibition of PAD4 activity by a novel therapeutic, BMS-P5, abolished citrullination of histone H3 and NET releases, thus improving the disease prognosis in patients and mice with myeloma [91]. The PAD4 inhibitor Cl-amidine significantly reduced NET formation, the number of breast cancer cells that extravasated into the lung tissue, however, was not altered [59]. Moreover, another PAD4 inhibitor, GSK484, was recently shown to prevent tumor-associated renal dysfunction in mice and the effect was determined to be NET-mediated [23]. Targeting PAD4 has been well acknowledged to have anti-NET capacity and an anti-tumor effect, although the exact molecular mechanisms of long-term therapy with PAD4 inhibitors and their long-term effects need further studies. Alternatively, the disruption of NET formation can be achieved by targeting the receptor for G-CSF (G-CSFR). A recent report by Wang et al. [92] underlined the ability of anti-G-CSFR monoclonal antibodies to inhibit NET release, as well as to downregulate hyperinflammatory reactions in the course of infections with no impact on pathogen clearance. Thus, blocking the G-CSFR receptor might represent a promising option to treat NET-dependent conditions without compromising the immune response against pathogens [92]. Another therapeutic intervention may be based on the above-described targeting of transmembrane DNA receptor CCDC25, which thus decreases cancer invasiveness [60].

The specialists agree that Ca2+ signaling constitutes an important component of the process of NET formation [2,3]. In addition, several authors have claimed that Ca2+ influxes have pro-oncogenic impact [49,50,51]. Ca2+ influxes in physiological processes can come from two sources: intracellular Ca2+ stores and external Ca2+ entering across the plasma membrane through cell membrane channels [22]. Another therapeutic option in cancer is targeting the ability of NETs to secrete the Ca2+-binding proteins S100A8 and S100A9 [93]. Both proteins S100A8/A9 are able to recruit tumor cells, to maintain inflammatory milieu, promote tumor progression, and create a favorable environment for metastatic niche formation, although the molecular mechanisms underlying their involvement in these processes remain unknown [22,94,95,96]. Schenten et al. demonstrated that S100A8 and S100A9 are key players in the cancer progression and proposed further investigations, enabling the development of an appropriate therapeutic intervention [93]. Animal studies have shown that DNase [36,62,97], cathepsin C (CTSC) inhibitors [98], PAD4 inhibitors [59], and NE inhibitors [62,97] displayed certain anti-metastasis effects by abrogation of NET formation. DNase I treatment suppressed the development of gross metastases and the growth of established liver micrometastases in colorectal cancer animal models [36]. The previously mentioned interventions hindering NET formation, such as DNase, NE inhibitors, and PAD4 knockout, reduced spontaneous lung and liver metastasis of lung carcinoma cells in NET-deficient mice [97]. Inhibiting CTSC by a second-generation inhibitor AZD7986, a potential drug for neutrophil-mediated inflammatory diseases, effectively wrecked NETs and abrogated lung metastasis of breast cancer in murine model, though without significant influence on primary tumor growth [98,99]. In addition, metformin, a well know anti-diabetic drug, is also proposed for cancer treatment, but its mechanism of action is not completely understood. A recent clinical study uncovered anti-NET properties of metformin, predominantly dependent on the inhibitory effect of metformin on the protein kinase C (PKC)-NADPH oxidase pathway [100]. Moreover, hydroxychloroquine can impede NET formation, potentially modulating the upstream signaling pathway for autophagy [101]. Clinical data from pancreatic cancer patients showed that hydroxychloroquine downregulates hypercoagulability and decreases the rate of thromboembolic complications typically attributed to overproduction of NETs [43].

The above-mentioned findings support the potential of NET-targeting approaches for the decreasing of metastatic behavior of cancer cells and the boosting of efficiency of anti-cancer therapy. Targeting NETs is a tempting opportunity and worthwhile strategy, however the risk of severe infections in NET-depleted patients may limit its clinical applications and further studies are warranted to investigate this issue. Nevertheless, the potential benefits of blocking pro-tumorigenic TAN properties encourage further research. Both options—either to dismantle formed NETs or to block their production—require further testing. Such strategies and underlying molecular mechanisms are in their infancy and further data to explore their therapeutic potential and lack of severe side-effects are awaited [22].

## 7. Conclusions

The NETs exert numerous pro-tumorigenic effects at various steps of tumor development. A better understanding of the crosstalk between cancer and NETs can help to elucidate basic aspects of the immune response to cancer and to devise novel therapeutic interventions that can block cancer evasion mechanisms and prevent metastatic spread.

## Data Availability

No new data were created or analyzed in this study.

## References

[1] Teng T.S., Ji A.L., Ji X.Y., Li Y.Z. (2017). Neutrophils and Immunity: From Bactericidal Action to Being Conquered. J. Immunol. Res..

[2] Pruchniak M.P., Demkow U. (2019). Potent NETosis inducers do not show synergistic effects in vitro. Cent. Eur. J. Immunol..

[3] Manda-Handzlik A., Bystrzycka W., Cieloch A., Glodkowska-Mrowka E., Jankowska-Steifer E., Heropolitanska-Pliszka E., Skrobot A., Muchowicz A., Ciepiela O., Wachowska M. (2020). Nitric oxide and peroxynitrite trigger and enhance release of neutrophil extracellular traps. Cell Mol. Life Sci..

[4] Manda-Handzlik A., Fiok K., Cieloch A., Heropolitanska-Pliszka E., Demkow U. (2020). Convolutional Neural Networks-Based Image Analysis for the Detection and Quantification of Neutrophil Extracellular Traps. Cells.

[5] Leliefeld P.H.C., Koenderman L., Pillay J. (2015). How Neutrophils Shape Adaptive Immune Responses. Front. Immunol..

[6] Pruchniak M.P., Ostafin M., Wachowska M., Jakubaszek M., Kwiatkowska B., Olesinska M., Zycinska K., Demkow U. (2019). Neutrophil extracellular traps generation and degradation in patients with granulomatosis with polyangiitis and systemic lupus erythematosus. Autoimmunity.

[7] Becker R.C. (2020). COVID-19-associated vasculitis and vasculopathy. J. Thromb. Thrombolysis.

[8] Gomez-Moreno D., Adrover J.M., Hidalgo A. (2018). Neutrophils as effectors of vascular inflammation. Eur. J. Clin. Investig..

[9] Velten L., Haas S.F., Raffel S., Blaszkiewicz S., Islam S., Hennig B.P., Hirche C., Lutz C., Buss E.C., Nowak D. (2017). Human haematopoietic stem cell lineage commitment is a continuous process. Nat. Cell Biol..

[10] Bremnes R.M., Dønnem T., Al-Saad S., Al-Shibli K., Andersen S., Sirera R., Camps C., Marinez I., Busund L.T. (2011). The Role of Tumor Stroma in Cancer Progression and Prognosis: Emphasis on Carcinoma-Associated Fibroblasts and Non-small Cell Lung Cancer. J. Thorac. Oncol..

[11] Mishalian I., Granot Z., Fridlender Z.G. (2017). The diversity of circulating neutrophils in cancer. Immunobiology.

[12] Masucci M.T., Minopoli M., Carriero M.V. (2019). Tumor Associated Neutrophils. Their Role in Tumorigenesis, Metastasis, Prognosis and Therapy. Front. Oncol..

[13] Yang P., Li Y., Xie Y., Liu Y. (2019). Different Faces for Different Places: Heterogeneity of Neutrophil Phenotype and Function. J. Immunol. Res..

[14] Lee J., Lee D., Lawler S., Kim Y. (2021). Role of neutrophil extracellular traps in regulation of lung cancer invasion and metastasis: Structural insights from a computational model. PLoS Comput. Biol..

[15] Shaul M.E., Fridlender Z.G. (2018). Cancer related circulating and tumor-associated neutrophils—Subtypes, sources and function. FEBS J..

[16] Szczerba B.M., Castro-Giner F., Vetter M., Krol I., Gkountela S., Landin J., Scheidmann M.C., Donato C., Scherrer R., Singer J. (2019). Neutrophils escort circulating tumour cells to enable cell cycle progression. Nature.

[17] Wculek S.K., Malanchi I. (2015). Neutrophils support lung colonization of metastasis-initiating breast cancer cells. Nature.

[18] Berger-Achituv S., Brinkmann V., Abed U.A., Kühn L.I., Ben-Ezra J., Elhasid R., Zychlinsky A. (2013). A proposed role for neutrophil extracellular traps in cancer immunoediting. Front. Immunol..

[19] Demers M., Krause D.S., Schatzberg D., Martinod K., Voorhees J.R., Fuchs T.A., Scadden D.T., Wagner D.D. (2012). Cancers predispose neutrophils to release extracellular DNA traps that contribute to cancer-associated thrombosis. Proc. Natl. Acad. Sci. USA.

[20] Demers M., Wagner D.D. (2013). Neutrophil extracellular traps: A new link to cancer-associated thrombosis and potential implications for tumor progression. Oncoimmunology.

[21] Nie M., Yang L., Bi X., Wang Y., Sun P., Yang H., Liu P., Li Z., Xia Y., Jiang W. (2019). Neutrophil Extracellular Traps Induced by IL8 Promote Diffuse Large B-cell Lymphoma Progression. Clin. Cancer Res..

[22] Tolle F., Umansky V., Utikal J., Kreis S., Bréchard S. (2021). Neutrophils in Tumorigenesis: Missing Targets for Successful Next Generation Cancer Therapies?. Int. J. Mol. Sci..

[23] Cedervall J., Zhang Y., Huang H., Zhang L., Femel J., Dimberg A., Olsson A.K. (2015). Neutrophil extracellular traps accumulate in peripheral blood vessels and compromise organ function in tumor-bearing animals. Cancer Res..

[24] Li Y., Yang Y., Gan T., Zhou J., Hu F., Hao N., Yuan B., Chen Y., Zhang M. (2019). Extracellular RNAs from lung cancer cells activate epithelial cells and induce neutrophil extracellular traps. Int. J. Oncol..

[25] Mitroulis I., Kambas K., Chrysanthopoulou A., Skendros P., Apostolidou E., Kourtzelis I., Drosos G.I., Boumpas D.T., Ritis K. (2011). Neutrophil extracellular trap formation is associated with IL-1beta and autophagy-related signaling in gout. PLoS ONE.

[26] Gupta A.K., Joshi M.B., Philippova M., Erne P., Hasler P., Hahn S., Resink T.J. (2010). Activated endothelial cells induce neutrophil extracellular traps and are susceptible to NETosis-mediated cell death. FEBS Lett..

[27] Tadie J.M., Bae H.B., Jiang S., Park D.W., Bell C.P., Yang H., Pittet J.F., Tracey K., Thannickal V.J., Abraham E. (2013). HMGB1 promotes neutrophil extracellular trap formation through interactions with Toll-like receptor 4. Am. J. Physiol. Lung Cell Mol. Physiol..

[28] Ng H., Havervall S., Rosell A., Aguilera K., Parv K., von Meijenfeldt F.A., Lisman T., Mackman N., Thålin C., Phillipson M. (2021). Circulating Markers of Neutrophil Extracellular Traps Are of Prognostic Value in Patients with COVID-19. Arterioscler. Thromb. Vasc. Biol..

[29] Oklu R., Sheth R.A., Wong K.H.K., Jahromi A.H., Albadawi H. (2017). Neutrophil extracellular traps are increased in cancer patients but does not associate with venous thrombosis. Cardiovasc. Diagn. Ther..

[30] Seo J.D., Gu J.-Y., Jung H.S., Kim Y.J., Kim H.K. (2019). Contact system activation and neutrophil extracellular trap markers: Risk factors for portal vein thrombosis in patients with hepatocellular carcinoma. Clin. Appl. Thromb. Hemost..

[31] Rosell A., Aguilera K., Hisada Y., Schmedes C., Mackman N., Wallén H., Lundström S., Thålin C. (2021). Prognostic value of circulating markers of neutrophil activation, neutrophil extracellular traps, coagulation and fibrinolysis in patients with terminal cancer. Sci. Rep..

[32] Saffarzadeh M., Juenemann C., Queisser M.A., Lochnit G., Barreto G., Galuska S.P., Lohmeyer J., Preissner K.T. (2012). Neutrophil extracellular traps directly induce epithelial and endothelial cell death: A predominant role of histones. PLoS ONE.

[33] Cedervall J., Hamidi A., Olsson A.-K. (2018). Platelets, NETs and cancer. Thromb. Res..

[34] Schedel F., Mayer-Hain S., Pappelbaum K.I., Metze D., Stock M., Goerge T., Loser K., Sunderkötter C., Luger T.A., Weishaupt C. (2020). Evidence and impact of neutrophil extracellular traps in malignant melanoma. Pigment. Cell Melanoma Res..

[35] Richardson J.J.R., Hendrickse C., Gao-Smith F., Thickett D.R. (2017). Neutrophil extracellular trap production in patients with colorectal cancer in vitro. Int. J. Inflam..

[36] Tohme S., Yazdani H.O., Al-Khafaji A.B., Chidi A.P., Loughran P., Mowen K., Wang Y., Simmons R.L., Huang H., Tsung A. (2016). Neutrophil extracellular traps promote the development and progression of liver metastases after surgical stress. Cancer Res..

[37] Yazdani H.O., Roy E., Comerci A.J., van der Windt D.J., Zhang H., Huang H., Loughran P., Shiva S., Geller D.A., Bartlett D.L. (2019). Neutrophil extracellular traps drive mitochondrial homeostasis in tumors to augment growth. Cancer Res..

[38] Kanamaru R., Ohzawa H., Miyato H., Matsumoto S., Haruta H., Kurashina K., Saito S., Hosoya Y., Yamaguchi H., Yamashita H. (2018). Low density neutrophils (LDN) in postoperative abdominal cavity assist the peritoneal recurrence through the production of neutrophil extracellular traps (NETs). Sci. Rep..

[39] Thålin C., Lundström S., Seignez C., Daleskog M., Lundström A., Henriksson P., Helleday T., Phillipson M., Wallén H., Demers M. (2018). Citrullinated histone H3 as a novel prognostic blood marker in patients with advanced cancer. PLoS ONE.

[40] Lima L.G., Monteiro R.Q. (2013). Activation of blood coagulation in cancer: Implications for tumour progression. Biosci. Rep..

[41] Jung H.S., Gu J., Kim J.-E., Nam Y., Song J.W., Kim H.K. (2019). Cancer cell-induced neutrophil extracellular traps promote both hypercoagulability and cancer progression. PLoS ONE.

[42] Gervaso L., Dave H., Khorana A.A. (2021). Venous and Arterial Thromboembolism in Patients with Cancer. JACC CardioOncol..

[43] Boone B.A., Murthy P., Miller-Ocuin J., Doerfler W.R., Ellis J.T., Liang X., Ross M.A., Wallace C.T., Sperry J.L., Lotze M.T. (2018). Chloroquine reduces hypercoagulability in pancreatic cancer through inhibition of neutrophil extracellular traps. BMC Cancer.

[44] Phan T.G., Croucher P.I. (2020). The dormant cancer cell life cycle. Nat. Rev. Cancer.

[45] Albrengues J., Shields M.A., Ng D., Park C.G., Ambrico A., Poindexter M.E., Upadhyay P., Uyeminami D.L., Pommier A., Kuttner V. (2018). Neutrophil extracellular traps produced during inflammation awaken dormant cancer cells in mice. Science.

[46] Orgaz J., Pandya P., Dalmeida R., Karagiannis P., Sanchez-Laorden B., Viros A., Albrengues J., Nestle F.O., Ridley A.J., Gaggioli C. (2014). Diverse matrix metalloproteinase functions regulate cancer amoeboid migration. Nat. Commun..

[47] Sargiannidou I., Qiu C., Tuszynski G.P. (2004). Mechanisms of thrombospondin-1-mediated metastasis and angiogenesis. Semin. Thromb. Hemost..

[48] Sanz-Moreno V., Balkwill F.R. (2018). Mets and NETs: The Awakening Force. Immunity.

[49] Hoenicke L., Zender L. (2012). Immune surveillance of senescent cells–biological significance in cancer- and non-cancer pathologies. Carcinogenesis.

[50] Shankaran V., Ikeda H., Bruce A.T., White J.M., Swanson P.E., Old L.J., Schreiber R.D. (2001). IFNgamma and lymphocytes prevent primary tumour development and shape tumour immunogenicity. Nature.

[51] Chitadze G., Lettau M., Bhat J., Wesch D., Steinle A., Furst D., Mytilineos J., Kalthoff H., Janssen O., Oberg H.H. (2013). Shedding of endogenous MHC class I-related chain molecules A and B from different human tumor entities: Heterogeneous involvement of the a disintegrin and metalloproteases 10 and 17. Int. J. Cancer.

[52] Krotova K., Khodayari N., Oshins R., Aslanidi G., Brantly M.L. (2020). Neutrophil elastase promotes macrophage cell adhesion and cytokine production through the integrin-Src kinases pathway. Sci. Rep..

[53] Cho H., Matsumoto S., Fujita Y., Kuroda A., Menju T., Sonobe M., Kondo N., Torii I., Nakano T., Lara P.N. (2017). Trametinib plus 4-methylumbelliferone exhibits antitumor effects by ERK blockade and CD44 downregulation and affects PD-1 and PD-L1 in malignant pleural mesothelioma. J. Thorac. Oncol..

[54] Onuma A., He J., Xia Y., Zhang H., Genkin D., Tetz G., Huang H. (2020). and Tsung, A. Neutrophil extracellular traps blockade in combination with PD-1 inhibition in treatment of colorectal cancer metastasis. J. Clin. Oncol..

[55] Leach J., Morton J.P., Sansom O.J. (2019). Neutrophils: Homing in on the myeloid mechanisms of metastasis. Mol. Immunol..

[56] Winkler J., Abisoye-Ogunniyan A., Metcalf K.J., Werb Z. (2020). Concepts of extracellular matrix remodelling in tumour progression and metastasis. Nat. Commun..

[57] Brostjan C., Oehler R. (2020). The role of neutrophil death in chronic inflammation and cancer. Cell Death Discov..

[58] Das A., Monteiro M., Barai A., Kumar S., Sen S. (2017). MMP Proteolytic Activity Regulates Cancer Invasiveness by Modulating Integrins. Sci. Rep..

[59] Park J., Wysocki R.W., Amoozgar Z., Maiorino L., Fein M.R., Jorns J., Schott A.F., Kinugasa-Katayama Y., Lee Y., Won N.H. (2016). Cancer cells induce metastasis-supporting neutrophil extracellular DNA traps. Sci. Transl. Med..

[60] Yang L., Liu Q., Zhang X., Liu X., Zhou B., Chen J., Huang D., Li J., Li H., Chen F. (2020). DNA of Neutrophil Extracellular Traps Promotes Cancer Metastasis via CCDC25. Nature.

[61] Kolaczkowska E., Jenne C.N., Surewaard B.G., Thanabalasuriar A., Lee W.Y., Sanz M.J., Mowen K., Opdenakker G., Kubes P. (2015). Molecular mechanisms of NET formation and degradation revealed by intravital imaging in the liver vasculature. Nat. Commun..

[62] Cools-Lartigue J., Spicer J., McDonald B., Gowing S., Chow S., Giannias B., Bourdeau F., Kubes P., Ferri L. (2013). Neutrophil extracellular traps sequester circulating tumor cells and promote metastasis. J. Clin. Investig..

[63] Najmeh S., Cools-Lartigue J., Rayes R.F., Gowing S., Vourtzoumis P., Bourdeau F., Giannias B., Berube J., Rousseau S., Ferri L.E. (2017). Neutrophil extracellular traps sequester circulating tumor cells via β1-integrin mediated interactions. Int. J. Cancer.

[64] Roth S., Agthe M., Eickhoff S., Möller S., Karsten C.M., Borregaard N., Solbach W., Laskay T. (2015). Secondary necrotic neutrophils release interleukin-16C and macrophage migration inhibitory factor from stores in the cytosol. Cell Death Discov..

[65] Monti M., De Rosa V., Iommelli F., Carriero M.V., Terlizzi C., Camerlingo R., Belli S., Fonti R., Di Minno G., Del Vecchio S. (2018). Neutrophil extracellular traps as an adhesion substrate for different tumor cells expressing RGD-binding integrins. Int. J. Mol. Sci..

[66] Reymond N., d’Agua B.B., Ridley A.J. (2013). Crossing the endothelial barrier during metastasis. Nat. Rev. Cancer.

[67] Kim Y., Stolarska M., Othmer H.G. (2007). A hybrid model for tumor spheroid growth in vitro I: Theoretical development and early results. Appl. Sci..

[68] Kim Y., Kang H., Powathil G., Kim H., Trucu D., Lee W., Lawler S., Chaplain M. (2018). Role of extracellular matrix and microenvironment in regulation of tumor growth and LAR-mediated invasion in glioblastoma. PLoS ONE.

[69] Kim Y., Powathil G., Kang H., Trucu D., Kim H., Lawler S., Chaplain M. (2015). Strategies of eradicating glioma cells: A multi-scale mathematical model with miR-451-AMPK-mTOR control. PLoS ONE.

[70] Kim Y., Othmer H.G. (2013). A hybrid model of tumor-stromal interactions in breast cancer. Bull. Math. Biol..

[71] Lee W., Lim S., Kim Y. (2017). The role of myosin II in glioma invasion: A mathematical model. PLoS ONE.

[72] Leal A.C., Mizurini D.M., Gomes T., Rochael N.C., Saraiva E.M., Dias M.S., Werneck C.C., Sielski M.S., Vicente C.P., Monteiro R.Q. (2017). Tumor-Derived Exosomes Induce the Formation of Neutrophil Extracellular Traps: Implications for the Establishment of Cancer-Associated Thrombosis. Sci. Rep..

[73] McInturff A.M., Cody M.J., Elliott E.A., Glenn J.W., Rowley J.W., Rondina M.T., Yost C.C. (2012). Mammalian target of rapamycin regulates neutrophil extracellular trap formation via induction of hypoxia-inducible factor 1 alpha. Blood.

[74] Yousefi S., Simon D., Stojkov D., Karsonova A., Karaulov A., Simon H.U. (2020). In vivo evidence for extracellular DNA trap formation. Cell Death Dis..

[75] Alfaro C., Teijeira A., Oñate C., Pérez G., Sanmamed M.F., Andueza M.P., Alignani D., Labiano S., Azpilikueta A., Rodriguez-Paulete A. (2016). Tumor-produced interleukin-8 attracts human myeloid-derived suppressor cells and elicits extrusion of neutrophil extracellular traps (NETs). Clin. Cancer.

[76] Paolillo M., Schinelli S. (2019). Extracellular Matrix Alterations in Metastatic Processes. Int. J. Mol. Sci..

[77] Masucci M.T., Minopoli M., Del Vecchio S., Carriero M.V. (2020). The Emerging Role of Neutrophil Extracellular Traps (NETs) in Tumor Progression and Metastasis. Front. Immunol..

[78] Martins-Cardoso K., Almeida V.H., Bagri K.M., Rossi M.I.D., Mermelstein C.S., König S., Monteiro R.Q. (2020). Neutrophil Extracellular Traps (NETs) Promote Pro-Metastatic Phenotype in Human Breast Cancer Cells through Epithelial–Mesenchymal Transition. Cancers.

[79] Krasnov G.S., Kudryavtseva A.V., Snezhkina A.V., Lakunina V.A., Beniaminov A.D., Melnikova N.V., Dmitriev A.A. (2019). Pan-Cancer Analysis of TCGA Data Revealed Promising Reference Genes for qPCR Normalization. Front. Genet..

[80] Zha C., Meng X., Li L., Mi S., Qian D., Li Z., Wu P., Hu S., Zhao S., Cai J. (2020). Neutrophil extracellular traps mediate the crosstalk between glioma progression and the tumor microenvironment via the HMGB1/RAGE/IL-8 axis. Cancer Biol. Med..

[81] Francescangeli F., Laura De Angelis M., Zeuner A. (2020). COVID-19: A potential driver of immune-mediated breast cancer recurrence?. Breast Cancer Res..

[82] Kolaczkowska E., Kubes P. (2013). Neutrophil recruitment and function in health and inflammation. Nat. Rev. Immunol..

[83] Tillack K., Breiden P., Martin R., Sospedra M. (2012). T Lymphocyte Priming by Neutrophil Extracellular Traps Links Innate and Adaptive Immune Responses. J. Immunol..

[84] Bilyy R., Fedorov V., Vovk V., Leppkes M., Dumych T., Chopyak V., Schett G., Herrmann M. (2016). Neutrophil Extracellular Traps Form a Barrier between Necrotic and Viable Areas in Acute Abdominal Inflammation. Front. Immunol..

[85] Shinde-Jadhav S., Mansure J.J., Rayes R.F., Marcq G., Ayoub M., Skowronski R., Kool R., Bourdeau F., Brimo F., Spicer J. (2021). Role of neutrophil extracellular traps in radiation resistance of invasive bladder cancer. Nat. Commun..

[86] Perez-Gracia J.L., Melero I. (2020). CXCR1 and CXCR2 Chemokine Receptor Agonists Produced by Tumors Induce Neutrophil Extracellular Traps that Interfere with Immune Cytotoxicity. Immunity.

[87] Kraft B., Kress H.G. (2005). Indirect CB2 receptor and mediator-dependent stimulation of human whole-blood neutrophils by exogenous and endogenous cannabinoids. J. Pharmacol. Exp. Ther..

[88] Mabou Tagn A., Marino F., Legnaro M., Luini A., Pacchetti B., Cosentino M. (2019). A novel Standardized Cannabis sativa L. extract and its constituent cannabidiol inhibit human polymorphonuclear leukocyte functions. Int. J. Mol. Sci..

[89] Munir H., Jones J.O., Janowitz T., Hoffmann M., Euler M., Martins C.P., Welsh S.J., Shields J.D. (2021). Stromal-driven and Amyloid β-dependent induction of neutrophil extracellular traps modulates tumor growth. Nat. Commun..

[90] Yuzhalin A.E., Gordon-Weeks A.N., Tognoli M.L., Jones K., Markelc B., Konietzny R., Fischer R., Muth A., O’Neill E., Thompson P.R. (2018). Colorectal cancer liver metastatic growth depends on PAD4-driven citrullination of the extracellular matrix. Nat. Commun..

[91] Li M., Lin C., Deng H., Strnad J., Bernabei L., Vogl D.T., Burke J.J., Nefedova Y. (2020). A Novel Peptidylarginine Deiminase 4 (PAD4) Inhibitor BMS-P5 Blocks Formation of Neutrophil Extracellular Traps and Delays Progression of Multiple Myeloma. Mol. Cancer Ther..

[92] Wang H., Aloe C., Wilson N., Bozinovski S. (2019). G-CSFR antagonism reduces neutrophilic inflammation during pneumococcal and influenza respiratory infections without compromising clearance. Sci. Rep..

[93] Schenten V., Plançon S., Jung N., Hann J., Bueb J.-L., Bréchard S., Tschirhart E.J., Tolle F. (2018). Secretion of the phosphorylated form of S100A9 from neutrophils is essential for the proinflammatory functions of extracellular S100A8/A9. Front. Immunol..

[94] Passey R.J., Xu K., Hume D.A., Geczy C. (1999). S100A8: Emerging functions and regulation. J. Leukoc. Biol..

[95] Hiratsuka S., Watanabe A., Sakurai Y., Akashi-Takamura S., Ishibashi S., Miyake K., Shibuya M., Akira S., Aburatani H., Maru Y. (2008). The S100A8-serum amyloid A3-TLR4 paracrine cascade establishes a pre-metastatic phase. Nat. Cell Biol..

[96] Ghavami S., Chitayat S., Hashemi M., Eshraghi M., Chazin W.J., Halayko A.J., Kerkhoff C. (2009). S100A8/A9: A Janus-faced molecule in cancer therapy and tumorgenesis. Eur. J. Pharmacol..

[97] Rayes R.F., Mouhanna J.G., Nicolau I., Bourdeau F., Giannias B., Rousseau S., Quail D., Walsh L., Sangwan V., Bertos N. (2019). Primary tumors induce neutrophil extracellular traps with targetable metastasis promoting effects. JCI Insight.

[98] Xiao Y., Cong M., Li J., He D., Wu Q., Tian P., Wang Y., Yang S., Liang C., Liang Y. (2020). Cathepsin C promotes breast cancer lung metastasis by modulating neutrophil infiltration and neutrophil extracellular trap formation. Cancer Cell.

[99] Palmer R., Maenpaa J., Jauhiainen A., Larsson B., Mo J., Russell M., Root J., Prothon S., Chialda L., Forte P. (2018). Dipeptidyl Peptidase 1 Inhibitor AZD7986 Induces a Sustained, Exposure-Dependent Reduction in Neutrophil Elastase Activity in Healthy Subjects. Clin. Pharm. Ther..

[100] Menegazzo L., Scattolini V., Cappellari R., Bonora B.M., Albiero M., Bortolozzi M., Romanato F., Ceolotto G., de Kreutzeberg S.V., Avogaro A. (2018). The antidiabetic drug metformin blunts NETosis in vitro and reduces circulating NETosis biomarkers in vivo. Acta Diabetol..

[101] Boone B.A., Orlichenko L., Schapiro N.E., Loughran P., Gianfrate G.C., Ellis J.T., Singhi A.D., Kang R., Tang D., Lotze M.T. (2015). The receptor for advanced glycation end products (RAGE) enhances autophagy and neutrophil extracellular traps in pancreatic cancer. Cancer Gene Ther..

